# Acute ingestion of beetroot juice increases exhaled nitric oxide in healthy individuals

**DOI:** 10.1371/journal.pone.0191030

**Published:** 2018-01-25

**Authors:** Juliet L. Kroll, Chelsey A. Werchan, David Rosenfield, Thomas Ritz

**Affiliations:** Southern Methodist University, Dallas, TX, United States of America; Forschungszentrum Borstel Leibniz-Zentrum fur Medizin und Biowissenschaften, GERMANY

## Abstract

**Background and objective:**

Nitric oxide (NO) plays an important role in the airways’ innate immune response, and the fraction of exhaled NO at a flow rate of 50mL per second (F_E_NO_50_) has been utilized to capture NO. Deficits in NO are linked to loss of bronchoprotective effects in airway challenges and predict symptoms of respiratory infection. While beetroot juice supplements have been proposed to enhance exercise performance by increasing dietary nitrate consumption, few studies have examined the impact of beetroot juice or nitrate supplementation on airway NO in contexts beyond an exercise challenge, which we know influences F_E_NO_50_.

**Methods:**

We therefore examined the influence of a beetroot juice supplement on F_E_NO_50_ in healthy males and females (n = 38) during periods of rest and in normoxic conditions. F_E_NO_50_, heart rate, blood pressure, and state affect were measured at baseline, 45 minutes, and 90 minutes following ingestion of 70ml beetroot juice (6.5 mmol nitrate). Identical procedures were followed with ingestion of 70ml of water on a control day.

**Results:**

After beetroot consumption, average values of the natural log of F_E_NO_50_ (lnF_E_NO_50_) increased by 21.3% (Cohen’s *d* = 1.54, p < .001) 45 minutes after consumption and by 20.3% (Cohen’s *d* = 1.45, p < .001) 90 min after consumption. On the other hand, only very small increases in F_E_NO_50_ were observed after consumption of the control liquid (less than 1% increase). A small subset (n = 4) of participants completed an extended protocol lasting over 3 hours, where elevated levels of F_E_NO_50_ persisted. No significant changes in cardiovascular measures were observed with this small single dose of beetroot juice.

**Conclusion:**

As NO serves a key role in innate immunity, future research is needed to explore the potential clinical utility of beetroot and dietary nitrate to elevate F_E_NO_50_ and prevent respiratory infection.

## Introduction

Nitric oxide (NO) is a molecule that plays an important role in the airway’s innate immune response in addition to a host of other functions including vasodilation and neurotransmission [1]. Three types of NO synthases (NOS) produce NO; inducible NOS (iNOS), endothelial NOS (eNOS), and neuronal NOS (nNOS). The fraction of NO in exhaled breath at a flow rate of 50mL per second (F_E_NO_50_) is utilized to capture levels of airway NO, presumed to be largely of iNOS origin in the bronchial epithelium [2;3]. Deficits in NO, in particular through reduced nNOS activity, are linked to the loss of bronchoprotective effects in airway challenges [4]. Lower levels of F_E_NO_50_ are observed in adult patients with cystic fibrosis [5] and have additionally predicted future symptoms of respiratory infection after stressful periods [6]. Conversely, the increased generation of NO in healthy individuals during human rhinovirus infections through multiple pathways is associated with fewer symptoms and more rapid viral clearance [7].

Research has largely focused on the pathway of endogenous NO production, where L-arginine is broken down by NOS. Beyond this conventional pathway, dietary nitrate is another source of NO. Circulating nitrate (NO_3_-) from both endogenous and dietary origin is converted to nitrite (NO_2_-) by reductase enzymes found in commensal bacteria in the oropharyngeal tract, gastrointentinal tract, trachea, and lower airway mucosal surfaces [8], which can be further reduced to NO in both blood and tissue [9]. Among the best sources of dietary nitrate is beetroot. Beetroot juice and dietary nitrate can enhance exercise performance and reduce long-term blood pressure in a dose-dependent response [10]. These findings are particularly pronounced in moments of hypoxia, where oxygen-dependent NOS enzyme activities are compromised and generation of NO must rely on the nitrate-nitrite conversion [9].

F_E_NO_50_ measurement guidelines recommend avoidance of nitrate-rich foods before assessments [11] as leafy greens and other nitrate-rich foods have been shown to elevate F_E_NO_50_ [12;13], but the exact time course and extent of the acute effects of more defined dosages of beetroot juice on F_E_NO_50_ are not well studied.

Exercise performance studies with beetroot juice have measured F_E_NO_50_ [14;15]; however, only in the context of exercise challenge, hypoxic environment, male-only samples, or smaller samples of trained athletes. Further research on the influence of nitrate-rich foods on nitric oxide is therefore indicated [16] to inform our understanding of the bioconversion of nitrate to nitric oxide. Because mood states are additionally implicated in F_E_NO_50_ changes, it would be important to also control for mood [1].

The primary aim of this proof-of-concept study was to generate understanding of the time period and degree to which one dose of beetroot juice can elevate F_E_NO_50_ in individuals of both genders while sedentary across periods of normoxia, and to determine whether these effects are sustained over an hour. The secondary aim was to determine if the mood state influences any changes observed in F_E_NO_50._ Taken together, these findings will inform future research exploring the utility of beetroot juice supplements on respiratory health.

## Materials and methods

### Participants

Volunteer undergraduate students, graduate students, and faculty members were recruited through a research subject pool and flyer advertisements on a university campus. Participants were non-smokers with no history of lung disease. Participants were instructed to refrain from exercising, eating, or drinking anything besides water for 1 hour before their session. Participants remained seated and were monitored in the lab space throughout duration of the session. Individuals with a history of alcohol abuse, illicit drug use, neurological disorders, and cardiovascular disorders, were excluded from participation, as were individuals who took antibiotics, or corticosteroids (oral or injected) in the last 2 months [11]. All procedures involving human participants were in accordance with the Southern Methodist University Institutional Review Board (# 2014-012-RITT) and with the 1964 Helsinki declaration and its later amendments. All participants provided written informed consent. Students were compensated with research credit towards course completion.

### Measures

F_E_NO_50_ was measured from steady exhale of the breath at a flow rate of 50 mL per second with the NIOX Mino (Aerocrine Systems, Solna, Sweden), a hand-held electrochemical analyzer, in parts per billion (ppb). The NIOX Mino has demonstrated high reliability in comparison with the “gold standard” chemiluminescence F_E_NO_50_ analyzer (NIOX) (r = 0.97) [17] and good test-retest reliability sufficient to recommend obtaining only one test value [17; 18]. The NIOX Mino has been observed to give readings slightly higher than the reference of the stationary NIOX analyzer with a mean difference of 1.2 ppb [17]. Heart rate (HR), systolic blood pressure (SBP), and diastolic blood pressure (DBP) were measured using the asuculatory method with an electronic upper arm blood pressure monitor, Omron M6 AC (Omron Healthcare, Hertogenbosch, Netherlands). Participants were seated in an upright chair with their legs uncrossed before the blood pressure monitor was placed on the participant’s left arm in order to assess one measurement of HR, SBP and DBP. Participants rested approximately 15 minutes before the first assessment, and were monitored in a seated position for the duration of each study visit.

Participant experience of momentary positive and negative affective state at each assessment was measured with the Positive and Negative Affect Schedule (PANAS), a 20-item self-report questionnaire consisting of a 10-item negative affect (NA) subscale and a 10-item positive affect (PA) subscale. Items are rated on a 5-point rating scale ranging from 0 = “very slightly/not at all” to 4 = “extremely,” and have displayed a high internal consistency (PA, Cronbach’s α = .89, NA, Cronbach’s α = .85) and test-retest reliability (PA, r = .68, NA, r = .71 [19]).

### Procedure

Participants visited the laboratory for both a control and experimental session. At the experimental session, 70ml of the beetroot juice Beet IT (98% concentrated beet juice, 2% lemon juice containing 400 mg/6.5 mmol nitrate)[20] was ingested following baseline measurements of F_E_NO_50_, HR, blood pressure, PA and NA. Additional measurements were conducted 45 and 90 minutes after consumption. Identical procedures were followed on the control day where participants consumed 70ml of water in lieu of beetroot juice. All assessments were conducted by two trained graduate students or an undergraduate research assistant with live supervision. Experimenters were not blinded to condition.

As participants served as their own control, sessions were conducted at the same time of day approximately one week apart and order of experimental versus control day was counterbalanced. Out of 38 participants who completed both sessions, six participants were tested outside of the one week window with 1, 2, 4, 5, 12, and 55 days between sessions. Average time between sessions was 8 days.

An extended protocol was conducted with a smaller number of participants with measurements taken 45, 90, 135 and 180 minutes following baseline for both experimental (beetroot) and control (water) days.

### Statistical analyses

First, ANCOVAs were used to compare baseline levels of all physiological parameters on experimental vs. control days. Then, repeated measures analyses of covariance (ANCOVA) were performed using the mixed effect models procedure within SPSS in order to test the effect of time and supplement condition (beetroot juice or water control) on airway, cardiovascular, and psychological parameters. The model included main effects of Time (pre-consumption of the drink, 45 minutes post-consumption, and 90 minutes post-consumption), Supplement (beetroot juice or water), Supplement X Time, Gender, Order of testing, and their interactions. Non-significant interactions were dropped in final models [21]. Compared to traditional repeated measures analysis of variance (ANOVA), mixed effects models (MEM) utilize all available data and avoid list-wise deletion of participants [22], thus increasing power and generalizability of results. Mixed effects models also do not assume sphericity, a central assumption of repeated ANCOVA, which is rarely met [21]. All available data was retained for all participants and included in analyses. F_E_NO_50_ was log transformed to reduce skewness. The log-transformed F_E_NO_50_ was used in all analyses of F_E_NO_50_. Cohen’s d was calculated to determine relative magnitude of change, where effect sizes of *d* = .8 indicates a large and *d* = 1.2 indicates a very large change [23]. As HR, SBP, and NA have been previously shown to influence F_E_NO_50_, each variable was tested as a time varying predictor of ln F_E_NO_50_ in both aggregated and disaggregated forms.

Post hoc power analyses, using the multilevel power analysis program PinT 2.12 [24] showed that we had greater than .80 power to detect an effect size larger than *d* = .64 (between a medium and large effect size).

## Results

### Participant characteristics at baseline

Our sample was comprised of 38 healthy subjects, 23 male and 15 female “Table 1.” Mean values of F_E_NO_50_, ln F_E_NO_50,_ HR, blood pressure, PA, and NA at each time point are shown in “Table 2.” Mixed model ANCOVAs demonstrated no significant differences in baseline levels of HR, blood pressure, nor ln F_E_NO_50_ between beetroot juice and control days.

**Table 1:** Participant demographics and physical characteristics.

	Mean	SD	Range
Age (years)	21	5.4	18–51
Height (cm)	170	10	105–200
Weight (kg)	66.7	12.8	46.3–112.5
BMI (kg/m^2^)	22.3	2.4	18.1–27.6
Gender n, %			
Male	23, 61%		
Female	15, 39%		
Race and Ethnicity n, %			
Caucasian Non-Hispanic	24, 63%		
Asian Non-Hispanic	5, 13%		
Caucasian Hispanic	4, 11%		
African-American Non-Hispanic	3, 8%		
Other Non-Hispanic	2, 5%		

**Table 2:** Cardiovascular, respiratory, and psychological measurements over time.

		Beetroot Session n = 35		Control Session n = 36	
		M	SD	M	SD
F_E_NO_50_ (ppb)	Pre	19.1	9.4	20.7	12.0
	45 Min	34.6	13.7	20.9	12.4
	90 Min	33.7	15.5	20.9	12.5
lnF_E_NO_50_	Pre	2.95	2.24	3.03	2.48
	45 Min	3.54	2.62	3.04	2.52
	90 Min	3.52	2.74	3.04	2.53
Systolic BP (mm/Hg)	Pre	110.2	13.9	110.1	11.7
	45 Min	111.1	10.1	108.1	9.0
	90 Min	108.9	11.1	110.9	9.1
Diastolic BP (mm/Hg)	Pre	73.9	8.2	72.0	5.6
	45 Min	75.9	8.8	71.2	5.7
	90 Min	75.3	7.9	75.6	9.6
Heart Rate (bpm)	Pre	69.9	10.3	68.8	8.2
	45 Min	69.4	10.5	66.0	9.3
	90 Min	69.9	11.6	67.3	13.3
PANAS-PA	Pre	9.5	6.0	9.6	5.8
	45 Min	7.6	5.7	7.0	4.8
	90 Min	6.5	5.9	6.1	4.6
PANAS-NA	Pre	5.5	3.4	4.9	3.9
	45 Min	4.7	3.5	3.9	3.5
	90 Min	3.4	3.1	3.3	2.7

Mixed model repeated measures ANOVAs were utilized to establish that there was no significant difference between baseline values in physiological variables on the experimental and control days at the p< .05 level. PANAS-PA and PANAS-NA reflect mean values of subscales. Two subjects did not attend the water control session, and three subjects did not attend the beetroot supplement session.

### Effect of beetroot juice on mood

Mixed model ANCOVA revealed a time effect for PA, *F*(4,167) = 9.54, *p <* .*001*, and NA, *F*(4,159) = 5.48, *p* < .001, indicating that both positive and negative affect decreased significantly over time. No effects were found for Supplement or Supplement x Time interaction.

As reductions in mood were observed in both conditions over time, PA and NA were included in an additional MEM as a time varying predictors of lnF_E_NO_50_. PA was not found to be related to lnF_E_NO_50_ across assessments (*p =* .21); however, the triple interaction of Time X Gender X NA was significant F(14,10) = 2.91, *p =* .048, on the beetroot juice day only, indicating average levels of NA for males after beetroot juice consumption was related to lnF_E_NO_50_ across assessments. Exploratory analyses revealed that those with an above average baseline NA demonstrated a blunted elevation in lnF_E_NO_50_ after beetroot juice compared to males low in baseline NA, who showed an exaggerated elevation in lnF_E_NO_50_. These effects were largely driven by one participant, who was low on NA and had a particularly large elevation in lnF_E_NO_50_ after beetroot juice consumption. There was no difference in baseline lnF_E_NO_50_ between the groups.

### Effect of beetroot juice on F_E_NO_50_

**Fig 1:**
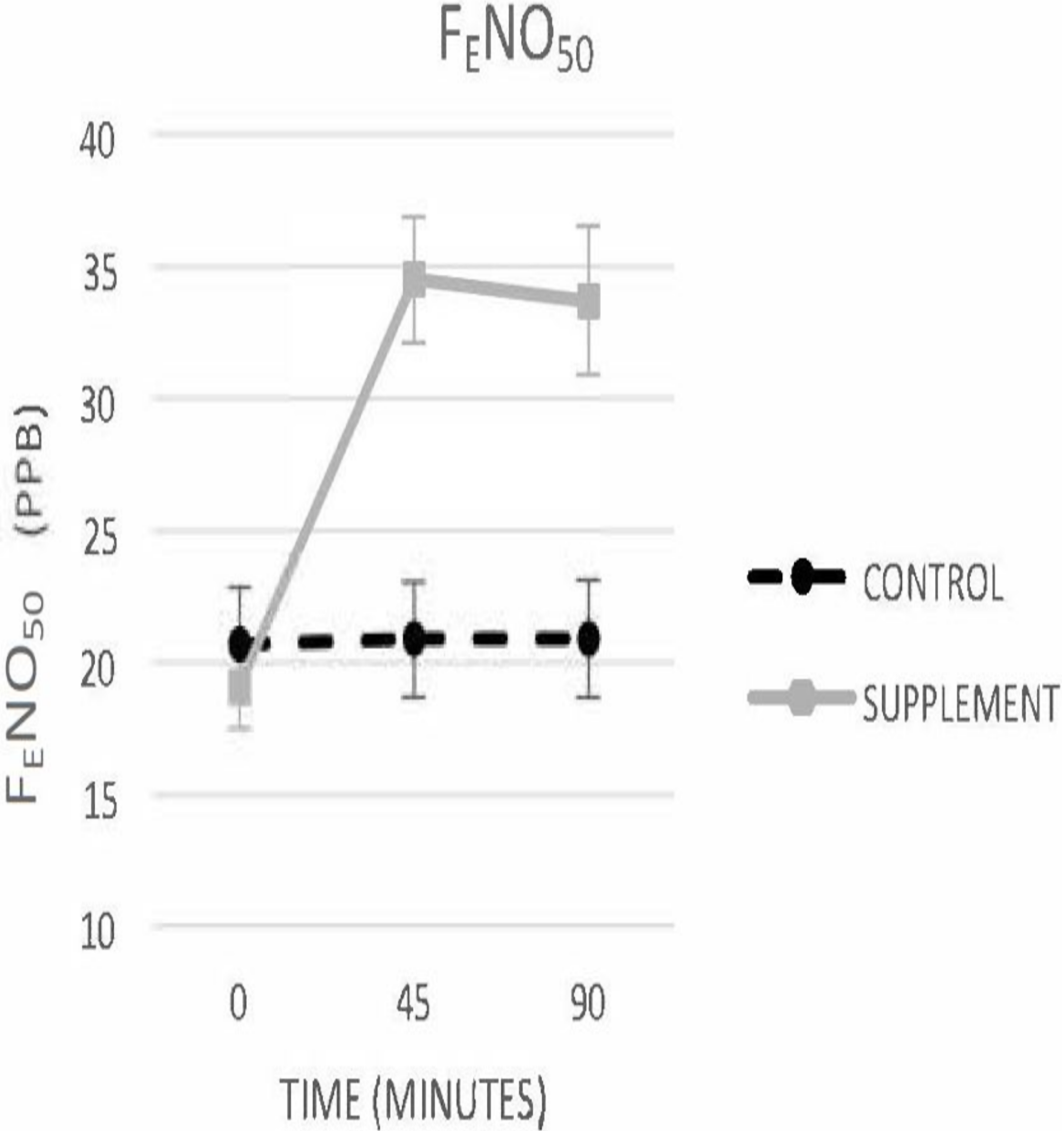
Average values of F_E_NO_50_ on both control and experimental days. Error bars indicate standard error.

Mixed model ANCOVA revealed a Supplement x Time interaction, F(2,27) = 51.60, *p*<. 001, indicating that the increase in lnF_E_NO_50_ over time was significantly greater when participants consumed beetroot juice than water. lnF_E_NO_50_ increased in all participants after beetroot juice consumption “Fig 1.” The overall pattern of change yielded a very large effect, with average lnF_E_NO_50_increasing by 21.3% (Cohen’s *d* = 1.54, p < .001) 45 minutes after consumption and by 20.3% (Cohen’s *d* = 1.45, p < .001) after 90 min. LnF_E_NO_50_ remained stable on the control day “Table 2”.

### Cardiovascular effect of beetroot juice

There were no significant differences observed over time or between treatment days for HR, SBP, or DBP after acute ingestion of beetroot juice, *p*>.05 (Table 2). As cardiovascular activity has previously been observed to influence lnF_E_NO_50_ independent of beetroot juice, HR and SBP were included in an additional MEM as time varying predictors of lnF_E_NO_50_. Neither were found to be related to lnF_E_NO_50_ across assessments.

### Extended protocol

**Fig 2:**
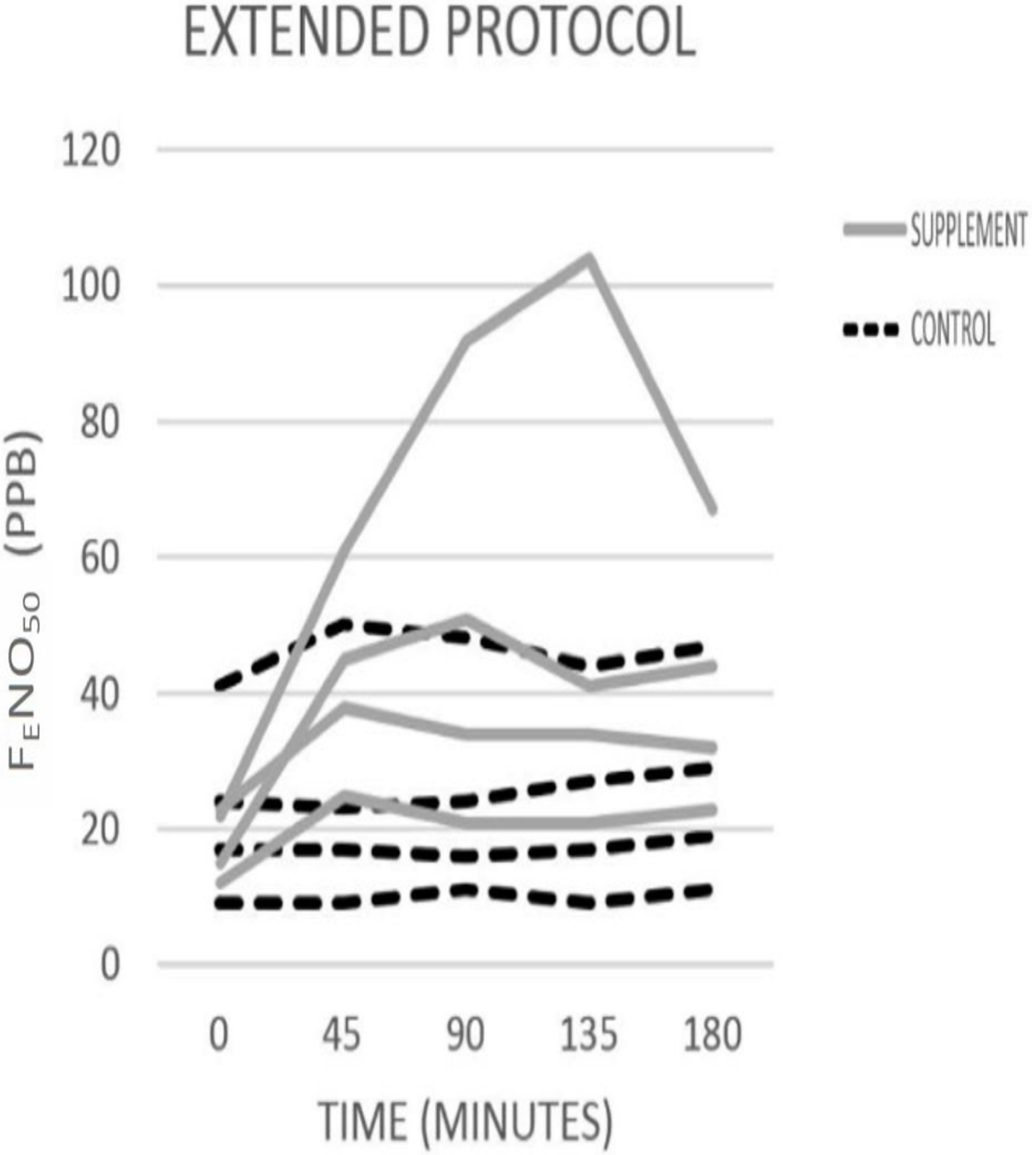
Raw values of F_E_NO_50_ on control and experimental days for extended protocol. Dashed black lines indicate water control day and solid grey lines indicates experimental day.

Only four individuals participated in the extended protocol (measuring F_E_NO_50_ up to 180 minutes after consumption). Given the small number of participants, no analyses of this extended data were performed. However, we present this data in a descriptive format to provide some preliminary information about the length of time for which F_E_NO_50_ remains elevated after consumption of the beetroot juice. On average F_E_NO_50_increased from baseline (mean = 18.0 ppb, SD = 5.4) by 119.4% or 21.5 ppb at 180 minutes after beetroot juice ingestion (mean = 39.5 ppb, SD = 20.0). After water consumption, F_E_NO_50_ (mean = 22.8 ppb, SD = 13.6) increased by 16.2% or 3.8 ppb at 180 minutes after water consumption (mean = 26.5 ppb, SD = 15.5) “Fig 2”.

## Discussion

Our study demonstrated robust increases in F_E_NO_50_ after ingestion of one well-defined dose of beetroot juice in a normoxic environment and independent of cardiovascular or psychological influences. F_E_NO_50_ increases were sustained for 90 minutes, and supplementary evidence suggests that increases likely persist for over 3 hours. These findings corroborate the utility of beetroot juice to elevate F_E_NO_50_, which we hypothesize is due to increased dietary nitrate in beetroot juice. Previous research has established an association among nitrate-rich foods and elevated F_E_NO_50_ [12;13]; however, the exact amount of F_E_NO_50_ increase achieved by the Beet It supplement and its time course in both genders, independent of hypoxia, psychological influence, or cardiovascular exertion has not, to the best of our knowledge, been previously determined.

F_E_NO_50_ is used clinically as a marker of allergic airway inflammation in asthma and increasingly in treatment decisions [25]; however, in health, F_E_NO_50_ is largely determined by iNOS activity in epithelial cells [2;3], part of the innate immune defense against pathogens [7]. While the biological role of F_E_NO_50_ changes is debated [1;26], decreased F_E_NO_50_ is linked to reduced protection against bronchoconstriction [4;27]. It is additionally observed in individuals with poor airway health, such as smokers, a likely result of the downregulation of NO-generating enzymes by cigarette smoke [28], and individuals with cystic fibrosis, where the thick mucus level is posited to inhibit diffusion of NO from the bronchial wall [5;29]. This study was designed to minimize the influence of BMI, gender, age, smoking, strong emotions, diet, and the presence of a respiratory tract infection which are all known to influence F_E_NO_50_ [30;1]; such influences can yield a F_E_NO_50_ change comparable to the F_E_NO_50_ increase observed after beetroot juice consumption in this study. In a large study designed to identify influences on F_E_NO_50_ and their clinical implications, Dressel and colleagues demonstrated that external influences on F_E_NO_50_ (e.g. respiratory tract infection, allergic status, and smoking) act homogenously and independently on Fe_NO_ [31]. We speculate that the influence of beetroot juice would yield an increase in F_E_NO_50,_ regardless of baseline values; however, the extent of this influence will need systematic study to identify if beetroot consumption has an independent and homogenous influence on F_E_NO_50_ or if specific biological considerations (e.g. atopy, smoking status) may interact with the beetroot juice yielding a differential influence on F_E_NO_50_.

Our findings of sustained F_E_NO_50_ increases after beetroot juice, in all likelihood through an alternative pathway of dietary nitrate conversion, justify further study of the potential long-term benefits of beetroot juice and dietary nitrate on airway health.

Studies additionally suggest that F_E_NO_50_ is susceptible to acute and prolonged psychological states [1]. Although ratings of both PA and NA were consistently reduced after ingestion of both beetroot juice and control, average values of NA varied with lnF_E_NO_50_ after consumption of beetroot juice for males only. Increases in lnF_E_NO_50_ after beetroot juice were greatest for those low in baseline NA; however, the effects were largely driven by one individual. These findings appear to confirm that psychological states may influence F_E_NO_50_ and should be measured in future studies.

Beetroot juice consumption can reduce SBP in just 3 hours [32] and dose-dependent responses in blood pressure are observed after 24 hours [10]. We observed no significant changes in blood pressure 90 minutes after ingestion, which may be consistent with the previously observed dose-dependent response, suggesting a higher dose is needed to observe cardiovascular changes in our design. Insufficient power due to smaller sample size likely limited our ability to detect changes in cardiovascular measures; however, these were not the primary focus of our study and the functional benefits of beetroot juice ingestion, in terms of improvements in exercise capacity, are likely to be expected only after multiple days of supplementation [33].

While our study was limited in sample size, the effects on F_E_NO_50_ were sufficiently strong and uniform. Although the introduction of dietary nitrate in beetroot juice is the most plausible cause for the increases in F_E_NO_50_, we cannot rule out that this change may be attributed to another dietary component of beetroot juice with our design. Future studies should use a placebo drink containing all similar ingredients without dietary nitrate.

We also cannot speak to the exact origin of NO in elevated F_E_NO_50_. Although the airway epithelium is thought to be a major contributor to F_E_NO_50_ in health, dietary conversion of nitrate likely took place in the salivary glands as well [8]. Regardless of the origin, elevated F_E_NO_50_ indicates that ingestion of beetroot juice leads to an increase in bioavailability of NO, which could be expected to boost innate immune defense in upper and/or lower airways, including the oropharyngeal tract, which forms a major entry way for pathogens. Although the bioavailability of NO is lower in the lungs than in the nasal cavities, where the anti-microbial effect of NO requires a higher concentration [30], any elevation in NO regardless of origin may already exert an anti-pathogenic influence. Additional plasma levels of NO would have aided interpreting the extent and biological influence on the observed increases in F_E_NO_50_ after beetroot juice consumption; however, multiple studies have found that plasma NO and F_E_NO_50_ demonstrate a similar pattern of change after nitrate consumption [10;13], and our study was focused on a noninvasive, ecologically valid measurement of beetroot juice effects without the confluence of a likely stronger emotional response to blood draw. Despite these limitations, this study provides first evidence of manipulation success of F_E_NO_50_ elevations achieved by one consumption of beetroot juice containing 400mg of dietary nitrate in a healthy sample under normoxic conditions, controlling for potential influences of mood states on F_E_NO_50_.

Our findings demonstrate that short-term elevations of F_E_NO_50_ can be achieved with one dose of beetroot juice, providing proof of manipulation success for future studies examining the potential beneficial effects of long-term beetroot juice and dietary nitrate supplementation on respiratory infection and illness. These results build upon previous research indicating the utility of the dietary nitrate pathway in NO formation, and future research is needed to consolidate our findings with larger and repeated doses of beetroot juice.
